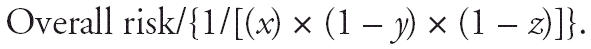# Comparing Risk of West Nile Virus against Risk of Adulticiding

**DOI:** 10.1289/ehp.114-a519a

**Published:** 2006-09

**Authors:** Steve Schofield, Martin Tepper, Janick Lalonde

**Affiliations:** Directorate of Force Health Protection, Canadian Forces Health Services, Group Headquarters, Department of National Defence, Ottawa, Ontario, Canada, E-mail: schofield.sw@forces.gc.ca

Peterson et al. (2006) compared the risk of ground-based ultra-low-volume (ULV) adulticiding against the risk of West Nile virus (WNV). They concluded that

[B]y virtually any current human-health measure, the risks from infection by WNV exceed the risks from exposure to mosquito insecticides. Therefore, perceptions that human-health risks from the insecticides used to control adult mosquitoes are greater than the risks from WNV currently cannot be supported by the current scientific evidence.

We appreciate their elegant analysis of health risks associated with residential exposure to ground-based ULV adulticides, and we concur that such risks are very low. However, we are concerned that their risk–risk comparison may be misinterpreted to indicate that the human health risk associated with adulticiding is more than offset by its potential for WNV disease reduction. Peterson et al. (2006) did not provide data to support this. Such a risk–benefit comparison requires at least two refinements.

First, it needs to take into account intervention effectiveness. Although it is not unreasonable to expect some benefit, it is unlikely that adulticiding is completely (or even mostly) effective. Hence, a risk–benefit comparison would need to address the likely situation of adulticiding being substantially < 100% effective, for example, by reducing estimates of adulticiding-based benefit by a factor of 1/*x,* where *x* represents the effectiveness of adulticiding.

Second, it needs to discount benefit based on upstream interventions. Adulticiding often takes place in the context of an integrated mosquito/WNV management program. In this situation, upstream approaches (e.g., larviciding, personal protection) discount the attributable benefit of downstream interventions (e.g., adulticiding). For example, use of larviciding and personal protection, respectively, providing *y* and *z* effectiveness, reduces the potential benefit of adulticiding by a factor of 1/[(1 − *y*) × (1 − *z*)].

Where upstream interventions are used and are fairly effective and adulticiding is not (or even if it is), adulticiding-attributable disease reduction may by substantially less than overall WNV risk. For example, if larviciding is 75% effective, personal protection 90% effective, and adulticiding 10% effective, the risk reduction achieved through adulticiding would be 1/400th of the overall risk of WNV-related disease; that is,